# Non-uptake of COVID-19 vaccines and reasons for non-uptake among healthcare workers in Uganda: a cross-sectional study

**DOI:** 10.1186/s12913-024-11137-2

**Published:** 2024-05-25

**Authors:** Nasimu Kyakuwa, Andrew Abaasa, Simon Mpooya, Hamza Kalutte, Christine Atuhairwe, Laurent Perez, Bernard Kikaire

**Affiliations:** 1 Uganda Virus Research Institute, Entebbe, Uganda; 2 MRC/UVRI & LSHTM Uganda Research Unit, Entebbe, Uganda; 3 Uganda Martyrs University, Kampala, Uganda; 4 Department of Medicine, Service of Immunology and Allergy, Centre for Human Immunology Lausanne, Lausanne University Hospital and University of Lausanne, Lausanne, Switzerland; 5 Makerere University College of Health Sciences, Kampala, Uganda

**Keywords:** COVID-19, Vaccine non-uptake, Healthcare workers, Determinants

## Abstract

**Background:**

Vaccines play a crucial role in eradicating and containing disease outbreaks. Therefore, understanding the reasons behind vaccine refusal and associated factors is essential for improving vaccine acceptance rates. Our objective was to examine the determinants of COVID-19 vaccine non-uptake and explore the reasons for non-uptake among healthcare workers (HCWs) in Uganda.

**Methods:**

Between July and August 2021, we conducted a cross-sectional study among healthcare workers in primary healthcare facilities (private and government) in Entebbe Municipality, Uganda. Participants were recruited using convenience sampling, and consenting individuals received credentials to access an electronic database and complete a structured questionnaire. There were no established HCWs contact registers in the municipality, and the study was conducted during a national lock down, therefore, the HCWs who were on duty at the time of the study were approached. The survey questions were based on the ‘3Cs’ model of vaccine hesitancy and focused on confidence, convenience, and complacency factors. Non-uptake of vaccines was defined as not having received any of the available vaccines in the country. We employed counts, percentages, and simple logit models to summarize the reasons for non-uptake of COVID-19 vaccines and to identify associated factors.

**Results:**

The study recruited 360 HCWs, 61.7% of whom were female, with an average age of 31 years (SD = 7.9). Among them, 124 (34.4%) healthcare workers did not receive any COVID-19 vaccine. Non-uptake of COVID-19 vaccines was independently associated with several factors, including age [35 + years adjusted odds ratio (aOR) = 0.30, 95% CI: 0.13–0.66 compared with 18–24 years], facility ownership [government, aOR = 0.22 (0.10–0.49) compared with private not-for-profit], previous testing for coronavirus [yes, aOR = 0.35 (0.19–0.65)], and previous involvement in COVID-19 vaccine activities [yes, aOR = 0.17 (0.10–0.29)]. The primary reasons cited for non-uptake of COVID-19 vaccines were related to a lack of confidence in the vaccines, such as concerns about side effects (79.8%) and the need for more time to understand the vaccines (89.5%), as well as the importance of weighing benefits and risks (84.7%) before being vaccinated. A smaller proportion, approximately 23%, cited reasons related to complacency and lack of convenience in accessing vaccination services.

**Conclusion:**

The high proportion of non-uptake of COVID-19 vaccines among this population primarily stems from a lack of confidence and trust in the vaccines, coupled with insufficient time allowed for users to make informed decisions. This underscores the urgent need for ongoing monitoring and trend analysis of vaccine non-uptake to guide the development and implementation of strategies aimed at building and sustaining vaccine confidence. Adequate time should be allowed to explain benefits of vaccination to the population to allay fears that might exist before actual vaccination is rolled out.

**Supplementary Information:**

The online version contains supplementary material available at 10.1186/s12913-024-11137-2.

## Background

The emergence of coronavirus disease 2019 [1] has presented a significant global health challenge, resulting in the loss of more than 7 million lives by December 31, 2023 [1]. Failing to effectively control the spread of the virus poses substantial risks to public health, including increased morbidity and mortality rates, which could overwhelm healthcare systems worldwide. In response, global efforts have prioritized vaccination as a crucial strategy to combat the pandemic. Immunization campaigns aim to achieve widespread immunity, thereby reducing the incidence of severe disease and hospitalization among the population.

Apart from the direct individual health benefits of conferring immunity, high vaccination coverage rates are important in attaining herd immunity [2, 3]. The attainment of herd immunity, which is critical for stopping disease spread, is directly affected by the non-uptake of COVID-19 vaccines [4].

On Friday 5th March 2021, the Uganda Ministry of Health received her first batch of 864,000 doses of AstraZeneca COVID-19 vaccine, shipped via the COVAX facility, and vaccination was prioritized for the following groups of people: health care workers, security personnel, teachers, journalists, persons aged 50 years and above and those with underlying health conditions [5]. The vaccines were freely accessible at no cost. The COVAX facility allocated 3,552,000 doses of the AstraZeneca vaccine to Uganda for the period of January–June 2021 [5]. On 31 July 2021, the country received 300,000 doses of the Sinovac vaccine from the Chinese government [6]. On 6th September 2021, 647,080 doses of Moderna vaccines were received, and on 21 September, 1,674,270 doses of Pfizer vaccine were received as donations from the US government [7]. On 8th October 2021, Uganda received the first batch of 196,000 doses of the Johnson and Johnson vaccine [8]. Other vaccines were subsequently introduced into the country. Hence, at the time of rollout of this study, only the AstraZeneca and Sinovac vaccines were available in designated public healthcare facilities in the country.

On the 10th March 2021, COVID-19 vaccines were launched [9] and prioritized for populations at high risk of developing severe disease and frontline healthcare workers (HCWs), who are not only at high risk of contracting the disease but also spreading the SARS-CoV-2 virus to patients under their care [10]. HCWs are a trusted source of health information and are likely to influence the use of COVID-19 vaccines [11]. Although HCWs are knowledgeable about the importance of vaccination, not all of them believe in vaccination, with some HCWs perceiving vaccines as unsafe and unnecessary [12, 13]. Studies have indicated that the non-uptake of COVID-19 vaccines among HCWs ranges from 4.3 to 72% [14] and varies with the role of HCWs, with nurses being less likely to take the vaccines [15–19].

Several sociodemographic factors, including age, may influence the use of COVID-19 vaccines among HCWs [20]. Younger age has been identified as one such factor associated with lower vaccine uptake, possibly attributed to the perception of a reduced risk of severe disease among younger individuals [21–23]. Other demographic factors, such as sex and education level, have been reported to affect the use of COVID-19 vaccines among healthcare workers [19, 21, 24].

The decision to get vaccinated immediately, delay of vaccination or complete refusal of vaccines may be influenced by factors such as confidence, complacency and convenience (‘3Cs’ model). The World Health Organization Strategic Advisory Group of Experts on Immunization (SAGE) working group developed the 3Cs model to explain vaccine hesitancy [25]. Confidence refers to a level of trust in the effectiveness and safety of vaccines, the delivery system, the reliability of health professionals, and the motivations of policymakers who make determinations about vaccines. Convenience refers to the degree to which the comfort, time, place, and quality of a vaccine affect the uptake of the vaccine, while complacency refers to a low perceived risk of vaccine-preventable diseases and therefore assumes that vaccination is not required to prevent the disease. The ‘3Cs’ model was later extended to 5Cs by adding calculations (extensive information searching by the individual) and communal orientation (considering collective responsibility) [25]. The decision to get vaccinated may vary with time, place, and type of vaccine; therefore, determinants of vaccine non-uptake need to be explored at different levels and among populations [26]. Vaccine confidence is one such important determinant of vaccine uptake. Trust in COVID-19 vaccines was affected by the fast-track production of the vaccines, which could have led to low uptake of the vaccines [27]. Also changes in government policies regarding vaccination mandates and vaccine distribution strategies to accommodate the need to conduct vaccinations among population groups at high risk of infection could affect the healthcare workers and/or public perception of COVID-19 vaccines [28]. A study by Alshareef et al., 2021 reported that 50.29% of healthcare workers were not willing to get vaccinated until the safety of the vaccines was demonstrated [24]. A similar study by Gadoth et al., (2021) reported a high COVID-19 vaccine non-uptake of 65.5% among HCWs in Los Angeles due to concerns about vaccine safety [29]. Vaccine convenience, which refers to the degree to which the comfort, time, place, and quality of a vaccine affect the uptake of the vaccine, is an important factor in determining vaccine uptake [13, 30, 31]. While numerous studies have explored individuals’ intentions to get vaccinated against COVID-19 once vaccines become available, there remains a significant gap in the literature regarding actual vaccine uptake among healthcare workers (HCWs). It is crucial to recognize that the intention to get vaccinated does not necessarily translate into actual uptake, emphasizing the importance of studying real-world vaccine acceptance and utilization among HCWs. A study by Nasimu et al., 2024 reported that 65.6% of the HCWs within primary healthcare facilities within Entebbe municipality took at least one dose of COVID-19 vaccine [32]. A significant proportion of HCWs, therefore, were hesitant to take the vaccines, and the reasons for non-uptake needed to be explored. Therefore, the aim of this study was to describe the determinants of COVID-19 vaccine non-uptake among HCWs in primary healthcare facilities within the Entebbe municipality. This study further explored the reasons for the non-uptake of vaccines among HCWs using the 3Cs + 2 model factors of vaccine hesitancy.

## Methods

### Study design

We conducted a cross-sectional study aimed at describing the non-uptake of COVID-19 vaccines and the associated reasons and factors among healthcare workers in private and government primary health facilities in Entebbe Municipality, Uganda.

### Study setting

The study was conducted in Entebbe Municipality, which is located approximately 40 km south of Kampala, the capital of Uganda. The estimated population (adults and children) in this municipality is approximately 700,000 people. There are about 40 healthcare facilities, approximately 80%, are privately owned. The municipality also has one regional referral hospital that was excluded from this study because a similar study was concurrently being conducted at this hospital.

### Study participants

Participants for this study were drawn from HCWs in the primary healthcare facilities described above. The participants were categorized as either medical or nonmedical staff. The medical HCWs included medical doctors, nurses, nursing assistants, paramedics, social workers, and research scientists, while the nonmedical HCWs included health center managers, accountants, receptionists, and janitors. Due to the absence of established healthcare workers’ contact lists within the municipality, participants were selected through convenience sampling. The study was also conducted during the national lockdown; therefore, only participants who were on duty at the time of data collection were approached.

### Data collection

The data were collected by research assistants between 1st July and 3rd August 2021. A structured questionnaire developed in Research Electronic Data Capture (REDCap) was used to collect the data. The questionnaire was adapted from the WHO Strategic Advisory Group on Experts (SAGE) on Immunization survey tool [33], for details, please refer to the supplementary material. The reasons for taking or not taking the vaccine were categorized based on the 3 C + 2 model of vaccine hesitancy, which includes factors such as confidence, complacency, convenience, collective responsibility and calculation. HCWs who agreed to participate and provided consent had the questionnaire link shared through email or WhatsApp. Participants who had no computer or smartphone were offered the study’s smartphone to complete the survey. The 3 C + 2 model of the reasons for the non-uptake of COVID-19 vaccines is provided in Table 1 below.

**Table 1:** 3 C + 2 model of reasons why HCWs were not vaccinated

*Confidence*
1. Did not think the vaccine was effective
2. Did not think the vaccine was safe
3. COVID-19 vaccine production was rushed
4. Had a bad experience or reaction with previous vaccines
5. Someone else told me he/she had/knows someone who had a bad reaction after vaccination
6. Concerned about side effects
*Complacency*
7. My job does not put me at a high risk of getting infected with corona virus
8. My age doesn’t put me at a high risk of severe COVID-19
9. There are better ways of prevention other than vaccination
10. Fear of needles
11. Did not think it was needed
12. COVID-19 is not so severe that I should get vaccinated
13. My immune system is so strong; it protects against disease
14. Bad experience with similar vaccination
*Lack of convenience*
15. Did not know where to get vaccination
16. Not possible to leave other work (home or office)
17. Long distance to the vaccination center
18. Transport costs to the vaccination center
19. Did not want to spend so much at the vaccination center
*Calculation (Increased information searching)*
20. Heard or read negative media
21. Did not know where to get good/reliable information
22. Distrust in government making the decision in my best interest
23. It’s important for me to fully understand COVID-19 vaccines before I get vaccinated
24. I closely consider whether COVID-19 vaccine is useful for me
25. I weigh the benefits and risks to make the best decision possible

### Statistical analysis

The data were electronically captured in the REDCap (Westlake, TX, USA) software database and transferred to STATA version 16 (Stata Corp, College Station, TX, USA) for statistical analysis. Participant characteristics were summarized overall and stratified by vaccine uptake status and compared using chi-square tests. Means with standard deviations and medians with interquartile ranges were used for continuous variables. The proportion of vaccine non-uptake was estimated as the number of participants who had not received any COVID-19 vaccine divided by the total number of participants studied, expressed as a percentage. The reasons for the lack of uptake of vaccines are summarized in the graphs. We used simple logistic regression models to determine factors associated with non-uptake of COVID-19 vaccines via univariate and multivariate models. We first fitted logit models for univariable analysis, and factors that attained a statistically significant likelihood ratio test (LRT) p value < 0.2 were considered for multivariable analysis. In the multivariable analysis, we used a backwards elimination approach, retaining factors that attained a statistically significant LRT p value < 0.05, with the exception of sex, which was included a priori. Before multivariable analysis, we checked for multicollinearity and assessed for inclusion only those factors that were more statistically significantly associated with non-uptake in the univariate analysis.

## Results

### Participant characteristics

We recruited 360 healthcare workers, mostly females (*n* = 222; 61.7%), with a mean age of 31 years (SD = 7.9). Approximately two-thirds were medical, with mostly a bachelor’s degree and above, *n* = 287 (80%), and the majority (*n* = 285; 79.2%) were aged more than 24 years (Table 2).

### Non-uptake of COVID-19 vaccines

A total of 124 (34.4%), 95%CI: 29.5-39.6% of the participants did not take up any COVID-19 vaccine despite free access. The percentage of individuals who did not take vaccines decreased with increasing age (42.7% in the 18–24 years age group vs. 39.2% in the 25–34 years age group and 20.2% in the 35 + year age group, *p* = 0.001); the percentage of individuals who did not take vaccines was greater among secondary contacts than among primary contacts (40.7% vs. 28.5%, *p* < 0.001), and the percentage of individuals taking vaccines from small roadside clinics (48.4%) was greater than among those in the Health Centre III & IV clinics (20.8%) and hospitals (29.8%, *p* < 0.001) (Table 2).

**Table 2:** Sociodemographic, clinical characteristics, and vaccine uptake among 360 healthcare workers enrolled in a COVID-19 uptake study in Uganda, 2021

Characteristics		Vaccine uptake		
	Total *N* = 360)	No (*n* = 124)	Yes(*n* = 236)	*p* values
**Gender**				
Male	222 (61.7)	71 (32.0)	151 (68.0)	0.212
Female	138 (38.3)	53 (38.4)	85 (61.6)	
**Age group (years)**				
18–24 Years	75(20.8)	32(42.7)	43(57.3)	0.001
25–34 Years	181(50.3)	71(39.2)	110(60.8)	
35 + Years	104(28.9)	21(20.2)	83(79.8)	
**Level of qualification**				
Certificate/Diploma	73(20.3)	29(39.7)	44(60.3)	0.288
Bachelors & Masters	287(79.7)	95(33.1)	192(66.9)	
**Job category**				
Medical	248 (68.9)	81 (32.7)	167 (67.3)	0.289
Nonmedical	112 (31.1)	43 (38.4)	69 (61.6)	
**Contacts(** ***n*** ** = 248)**				
Primary contacts**	151 (60.9)	43 (28.5)	108 (71.5)	< 0.001
Secondary contacts***	97 (39.1)	38 (40.7)	59 (59.3)	
**Level of service of the health facility**				
Hospitals	57 (15.8)	17 (29.8)	40 (70.2)	< 0.001
Health center III & IV	144 (40.0)	30 (20.8)	114 (79.2)	
Small Roadside clinics	159 (44.2)	77 (48.4)	82 (51.6)	
**Type of ownership**				
Private not for profit (PNFP)	84 (23.3)	39 (46.4)	45 (53.6)	< 0.001
Private for profit (PFP)	146 (40.6)	72 (49.3)	74 (50.7)	
Government	130 (36.1)	13 (10.0)	117 (90.0)	
**Previously cared for confirmed COVID-19 patient**				
No	226 (62.8)	70 (31.0)	156 (69.0)	0.072
Yes	134 (37.2)	54 (40.3)	80 (59.7)	
**Previously tested for Corona virus infection**				
No	89 (24.7)	58 (65.2)	31 (34.8)	< 0.001
Yes	271 (75.3)	66 (24.4)	205 (75.6)	
**Previous Corona test results (** ***N*** ** = 271)**				
Negative	230 (84.9)	53 (23.0)	177(77.0)	0.234
Positive	41 (15.1)	13 (31.7)	28 (68.3)	
**Ever involved in COVID-19 vaccine activities**				
No	150 (41.7)	91 (60.7)	59 (39.3)	< 0.001
Yes	210 (58.3)	33 (15.7)	177 (84.3)	

Note: p values-based Chi-square test; * significant at 5% level, ** HCWs who interface with patients first, ***HCWs who interface with patients who have been screened or deal with biological materials obtained from patients

### Factors associated with non-uptake of vaccines

According to the multivariable analysis, factors that were independently associated with non-uptake of the COVID-19 vaccine included age [25–34 years, adjusted odds ratio (aOR) = 0.7, 95% CI: 0.35–1.40, 35 + years aOR = 0.30, 95% CI: 0.13–0.66, all compared to 18–24 years], previous COVID-19 infection status [yes, aOR = 0.35, 95% CI: 0.19–0.65] and ever been involved in COVID-19 vaccine activities [yes, aOR = 0.17, 95% CI: 0.10–0.29]. Other factors are shown in Table 3 below.

**Table 3:** Sociodemographic and clinical factors associated with non-uptake of COVID-19 vaccines among healthcare workers in Uganda

Characteristics	uOR (95%CI)	LRT-pvalue	aOR (95%CI)	LRT-pvalue
**Gender**				0.185
Male	1.00	0.214	1.00	
Female	1.33 (0.85–2.07)		1.67 (0.95–2.94)	
**Age group (complete years)**		0.001		0.001
18–24	1.00		1.00	
25–34	0.87 (0.50–1.50)		0.70 (0.35–1.40)	
35+	0.34 (0.18–0.66)		0.30 (0.13–0.66)	
**Level of qualification**		0.291		
Certificate-diploma	1.00			
Bachelors +	0.75 (0.44–1.27)			
**Job category**				
Clinical	1.00	0.292		
Nonclinical	1.28 (0.81–2.04)			
**Contact level**				
Primary**	1.00	0.199		
Secondary***	1.34 (0.86–2.10)			
**Level of service of the health facility**		< 0.001		
Hospital	1.00			
Health center III & IV	0.62 (0.31–1.24)			
Small road side clinics	2.21 (1.16–4.22)			
**Type of ownership**		< 0.001		< 0.001
Private not for profit (PNFP)	1.00		1.00	
Private for profit (PFP)	1.12 (0.66–1.92)		0.90 (0.47–1.70)	
Government	0.13 (0.06–0.26)		0.22 (0.10–0.49)	
**Previously cared for a COVID-19 confirmed patient**		0.073		
No	1.00			
Yes	1.51 (0.96–2.35)			
**Previously tested for Corona virus infection**		< 0.001		< 0.001
No	1.00		1.00	
Yes	0.17 (0.11–0.29)		0.35 (0.19–0.65)	
**Previous Corona test results (** ***N*** ** = 271)**		0.005		
Negative	1.00			
Positive	2.06 (1.25–3.41)			
**Ever involved in COVID-19 vaccine activities**		< 0.001		< 0.001
No	1.00		1.00	
Yes	0.12 (0.07–0.20)		0.17 (0.10–0.29)	

Note: p values based Chi-square test; *_significant at 5% level, ** HCWs who interface with patients first, ***HCWs who interface with patients who have been screened or deal with biological materials obtained from patients

### Primary reasons why healthcare workers were not vaccinated

**Fig. 1:**
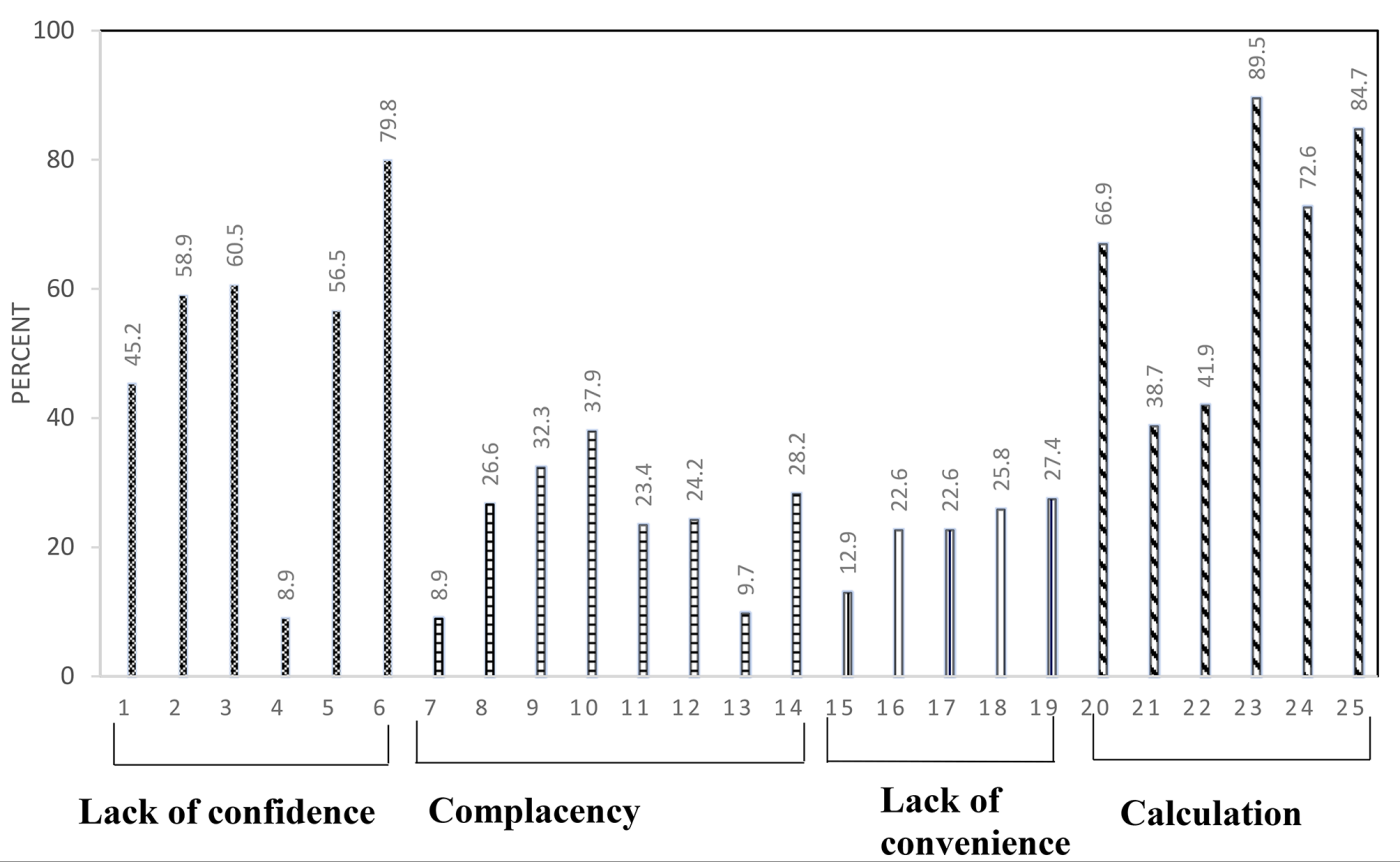
Reasons why HCWs were not vaccinated (*n* = 124)

The reasons for vaccine non-uptake are depicted in Fig. 1 below. Mostly, reasons related to lack of confidence in the vaccines, such as concerns about side effects (79.8%) and calculations, including insufficient time to understand the vaccines (89.5%) and weighing benefits and risk (84.7%) before being vaccinated, were considered key reasons for not receiving vaccination. An average of 23% of the respondents raised reasons related to complacency and lack of convenience (Fig. 1).

## Discussion

The findings of this study revealed that one in three healthcare workers did not take up COVID-19 vaccines despite their availability. This finding contrasts with that of Patrick et al., [34], who reported that one in ten healthcare workers in Uganda were unwilling to receive the COVID-19 vaccine [35]. The difference in findings may be attributed to differences in the study setting. Our study was conducted in an urban setting, while Patrick et al., conducted the study in a rural setting. It is also well known that vaccine non-uptake is context specific, varying from place to place, time to time and between populations [25]. Two-thirds of healthcare workers in the Patrick et al., study were nurses, and most were low-cadre healthcare workers; similar studies have reported low rates of COVID-19 vaccine uptake among nurses [15, 36]. Our study, however, didn’t report a significant relationship between the staff cadre and non-uptake of the vaccines. Globally, healthcare workers were among the priority groups for COVID-19 vaccination due to their increased risk of exposure to infections, and since they are a trusted source of information, non-uptake could have impacted the overall uptake of the vaccines by the general population. Furthermore, studies have shown that COVID-19 vaccine hesitant healthcare workers are less likely to recommend a COVID-19 vaccine to their patients [37].

Young age was associated with non-uptake of the vaccines, a finding that has been reported by similar studies [14, 15]. This finding is not surprising since higher morbidity and mortality rates due to COVID-19 have been reported among older patients [38, 39]. While being young is associated with a low risk of severe COVID-19, non-uptake of vaccines among this age group should be addressed. A study by James et al., 2021 that explored the factors associated with COVID-19 severity in US children and adolescents reported that 20% of the children admitted to the hospital suffered from severe disease [40], and being black was associated with greater disease severity. Furthermore, young individuals are highly mobile, which could contribute to increased transmission of the SARS-CoV-2 virus.

Access to vaccination services is a critical determinant of vaccine uptake. In this study, we found that working in a private health facility was associated with increased non-uptake of vaccines compared with working in government health facilities. In Uganda, the rollout of COVID-19 vaccination has been concentrated in government healthcare facilities. This inaccessibility to vaccination services could have led to higher non-uptake rates among HCWs in these facilities. Being a private HCW is also associated with stringent work schedules, and some HCWs (22.6%) reported not being able to leave their workplaces to go for vaccination. Inaccessibility to COVID-19 vaccines has been reported to be one of the barriers to COVID-19 vaccine uptake [41].

This study explored the effect of prior testing for coronavirus infection on vaccine uptake among HCWs. We found that HCWs who had never been tested for the coronavirus were less likely to take the vaccines than were the participants who had ever been tested. A study by Laura et al. [34] reported that 96% of participants consumed a COVID-19 vaccine at least once, mostly after infection with the coronavirus [42]. This difference in uptake could be due to differences in risk perceptions among HCWs. However, in our study, there was no relationship between the test results and the use of COVID-19 vaccines.

The study further revealed that healthcare workers who were not involved in COVID-19 vaccination-related activities were less likely to take up vaccines than those who were involved in vaccine-related healthcare. Healthcare workers who participate in vaccination services are trained about vaccines, which improves their understanding of vaccines, how they work and their safety, hence building confidence and trust in vaccination services. However, there is a paucity of data in this area; hence, further research is needed.

Using the 5 C constructs (confidence, convenience, complacency, calculation, and collective responsibility model) of the determinants of vaccine uptake, we found that a lack of confidence in vaccines and an increased search for information were associated with the non-uptake of vaccines. Greater than 50% of the healthcare workers did not take up the vaccines due to safety concerns, rushed vaccine production and concerns about side effects after vaccination. These concerns have also been reported by other studies as reasons for non-uptake of the vaccines [14, 35, 43, 44]. Shortly after the launch of COVID-19 vaccination in Uganda, safety concerns related to thromboembolism were reported about Oxford AstraZeneca vaccine [45], and this could have had an impact on the uptake of the vaccines by the HCWs. This study, however, didn’t explore individual safety concerns and their relationship with COVID-19 vaccines. Hence, vaccine confidence should be regularly monitored to detect new trends to prompt interventions to build and maintain vaccine confidence. More than two-thirds of the healthcare workers who never received the vaccines reported having read negative media about COVID-19 vaccines, needed more time to understand COVID-19 vaccines and weighed the benefits vs. the risks before deciding to receive the vaccines. This is not surprising, as COVID-19 vaccines were associated with many myths and misconceptions [46]. A systematic review of the studies done earlier during the first phase of the COVID-19 pandemic reported that 0.2–28.8% of the social media posts about the vaccines could be classified as misinformation [47]. Previously reported misconceptions about the vaccines included, the COVID-19 vaccine contains a microchip to control the population, the vaccine not having been tested on enough people, and getting infected with the virus after vaccination [46]. This indicates that information-seeking actions such as deciding to take the vaccine based on the sought or established reliable information were important determinants of vaccine uptake. Therefore, providing information that meets the expectations of the public is critical for one’s decision to vaccinate, specifically the trust that COVID‐19 vaccines are safe and effective.

This study is one of the few studies that has explored the reasons for non-uptake of COVID-19 vaccines among health care workers in sub-Saharan Africa. Information was collected during the peak of the epidemic, when the morbidity and mortality rates due to COVID-19 were highest. Therefore, the reasons for non-uptake would be most expressed during this time. Therefore, the findings of this study reflect true healthcare workers’ perceptions about COVID-19 vaccines. However, this study is limited by the fact that, we used convenience sampling; hence, the findings may not be generalizable to all healthcare workers. Only those HCWs who were on duty at the time of data collection were contacted and included in the study. Additionally, we cannot exclude reporting bias since we relied on self-reported information about vaccination.

## Conclusions

This study highlights a concerning level of vaccine non-uptake among HCWs in the Entebbe municipality, largely stemming from a lack of confidence and trust in the vaccines. This finding underscores the importance of continuous monitoring and trend identification to guide efforts aimed at building and sustaining vaccine confidence among HCWs. Given the association between participation in vaccine-related services and higher uptake, integrating vaccine safety information into continuous medical education programs for HCWs is essential to address safety concerns effectively. Moreover, it is imperative for government and development partners to ensure equitable involvement of both private and public healthcare systems in vaccination programs. This approach will help to enhance access to vaccines and strengthen overall vaccination coverage across the population.

This study revealed that a lack of confidence in vaccines among HCWs could influence their uptake by the general population since healthcare workers are a trusted source of information. Healthcare workers who do not trust vaccines are unlikely to recommend that their patients or population receive vaccines. Therefore, exploring the reasons for the non-uptake of vaccines should be an ongoing process, especially for new vaccines. Most government vaccine policies are based on well-known and studied diseases/infections, COVID-19 infections and vaccines provided the need to formulate vaccination policies in the face of an outbreak. Further research with qualitative approach is recommended to inform targeted interventions to address specific barriers to vaccine uptake among HCWs.

### Electronic supplementary material

Below is the link to the electronic supplementary material.

Supplementary Material 1

## Data Availability

Data cannot be shared publicly because of country-specific data sharing restrictions. Data are available from the UVRI Institutional Data Access/Ethics Committee (contact via +256773747607) for researchers who meet the criteria for access to confidential data.

## Abbreviations

COVID-19: Coronavirus Disease 2019
HCWs: Healthcare workers
LRT: Likelihood ratio test
SARS-CoV-2: Severe acute respiratory syndrome coronavirus-2
SAGE: Strategic Advisory Group on Experts
UVRI-REC: Uganda Virus Research Institute’s Research and Ethics Committee
WHO: World Health Organization
