# Supplementary material for: Non-uptake of COVID-19 vaccines and reasons for non-uptake among healthcare workers in Uganda: a cross-sectional study
Source: BMC Health Serv Res. 2024 May 25;24:663. doi: 10.1186/s12913-024-11137-2 (PMC11128104; doi:10.1186/s12913-024-11137-2)
Supplement: Supplementary file 1 — Supplementary Material 1 [file 12913_2024_11137_MOESM1_ESM.pdf]

DETERMINANTS AND LEVEL OF COVID-19 VACCINE UPTAKE AMONG HEALTHCARE WORKERS IN ENTEBBE MUNICIPALITY

Please complete the survey below.

Thank you!

Introduction

Acceptance of the COVID-19 vaccine will play a major role in combating the COVID-19 pandemic. Health workers (HCWs) were among the first group to receive vaccination in Uganda. In Entebbe municipality, the level of uptake and factors associated with uptake of COVID-19 vaccines are unknown. This study will describe the level of uptake and determinants associated with uptake of COVID-19 vaccine among HCWs in Entebbe municipality. Results of the study will inform future strategies on COVID-19 vaccination deployment and implementation among HCWs.
All data will be stored and processed in accordance with national regulations. Approvals for this study have been obtained from Uganda National Council for Science and Technology.
Approves for this survey have been obtained from UVRI Research Ethics Committee, and Uganda National Council for Science and Technology.

If you are willing to take part in this study, please confirm the following:

I understand that my participation is completely voluntary

- ◯ Yes
- ◯ No

I would like to take part in this study

- ◯ Yes
- ◯ No

Date of interview

____

Gender

- ◯ Female
- ◯ Male

Age

____
((Years))

Social Demographic Data

What is your level education?

- ◯ None
- ◯ Primary
- ◯ Secondary
- ◯ Diploma/Bachelors
- ◯ Masters
- ◯ PhD

Job category

- ◯ Medical
- ◯ Non-medical

What is your cadre/Job title(Medical)?	◯ Consultant/Senior Consultant/Professor<br>◯ Medical Officer Special Grade/Registrar<br>◯ Medical Officer/Dental Surgeon<br>◯ Intern Doctor<br>◯ Clinical officer/Paramedic<br>◯ Nursing officer/Midwife<br>◯ Laboratory technologist/technician<br>◯ Dental officer<br>◯ Other(Specify)<br>(Tick the most appropriate)
Others (please specify)	__________________________
What is your job title(Non-medical)?	◯ Receptionist<br>◯ Cashier<br>◯ Adminstrator<br>◯ Accountant<br>◯ Cleaner<br>◯ Security<br>◯ Driver<br>◯ Store keeper/Procurement<br>◯ Other (Specify)<br>(Tick the most appropriate)
Others (please specify)	__________________________
What is your area of operation?	◯ Out-patient<br>◯ Maternity/Antenatal<br>◯ In-patient wards<br>◯ Operating theatre<br>◯ Intensive Care Unit (ICU)<br>◯ Laboratory<br>◯ ART clinic<br>◯ Non-clinical area<br>◯ Administrative office<br>◯ Isolation ward/rooms<br>◯ Other (Specify)<br>(Tick the most appropriate)
Others (please specify)	__________________________
What is your level of service?	◯ Hospital<br>◯ Health centre IV<br>◯ Health centre III<br>◯ Health centre II<br>◯ Medical Centre<br>◯ Private Clinic<br>(please tick the most appropriate)
What is the type of ownership?	◯ Private not for profit<br>◯ Private for profit<br>◯ Government<br>(Tick the most appropriate)

Have you cared for patients infected with COVID-19?	◯ No<br>◯ Yes<br>◯ I don't know<br>(Tick the most appropriate)
Have you ever tested for COVID-19?	◯ Yes<br>◯ No<br>(Tick the most appropriate)
If yes above, what were the results?	◯ Negative<br>◯ Positive<br>◯ I did not receive results
Have you participated in any COVID-19 vaccine related activities?	◯ Yes<br>◯ No<br>(Tick the most appropriate)
Have you received COVID-19 vaccine?	◯ Yes<br>◯ No<br>(Tick the most appropriate)

If yes above, what were the reason(s)? (Provide an opinion for each by ticking the most appropriate.)

Confidence

1.Vaccines are safe	◯ Yes<br>◯ No
2. Encouragement by ministry of health	◯ Yes<br>◯ No
3. Vaccination was a requirement at workplace	◯ Yes<br>◯ No
4. COVID-19 vaccines are effective	◯ Yes<br>◯ No
5. Trust in the healthcare systems	◯ Yes<br>◯ No
6. Recommendation by a healthcare provider	◯ Yes<br>◯ No

Complacency

1. My health condition puts me at high risk of getting severe COVID-19	◯ Yes<br>◯ No
2. My age puts me at a high risk of getting severe COVID-19	◯ Yes<br>◯ No
3. My job puts me at a high risk of contracting the Corona virus	◯ Yes<br>◯ No
4. Someone i know died of COVID-19	◯ Yes<br>◯ No
5. Good experience with similar vaccination	◯ Yes<br>◯ No

Convenience

1. COVID-19 vaccine is free	◯ Yes<br>◯ No
2. Vaccination was near workplace/brought at workplace	◯ Yes<br>◯ No
3. My workplace created time to go for vaccination	◯ Yes<br>◯ No
4. Didn't have transport problem to the vaccination centre	◯ Yes<br>◯ No

Collective responsibility

1. Protect family, patients, and friends from getting infected	◯ Yes<br>◯ No
2. Recommendations by the friend/relative/religious leader	◯ Yes<br>◯ No
3. Vaccination is a collective action to prevent the spread of COVID-19	◯ Yes<br>◯ No
4. My friends/employer encouraged me to get vaccinated	◯ Yes<br>◯ No

If not vaccinated,what were the reason(s)? (Provide an opinion for each)

Confidence

1. Did not think the vaccine was effective	◯ Yes<br>◯ No
2. Did not think the vaccine was safe	◯ Yes<br>◯ No
3. COVID-19 vaccine production was rushed	◯ Yes<br>◯ No
4. Had a bad experience or reaction with previous vaccination	◯ Yes<br>◯ No
5. Someone else told me he/she had/knows someone who had a bad reaction after vaccination	◯ Yes<br>◯ No
6. Concerned about side effects	◯ Yes<br>◯ No
7. Had a bad experience with previous vaccinator/health clinic	◯ Yes<br>◯ No

Complaceny

1. My job doesn't put me at a high risk of getting infected with corona virus	◯ Yes<br>◯ No
2. My age doesn't put me at a high risk for severe COVID-19	◯ Yes<br>◯ No
3. There are better ways of prevention other than vaccination	◯ Yes<br>◯ No
4. Fear of needles	◯ Yes<br>◯ No
5. Did not think it was needed	◯ Yes<br>◯ No
6. COVID-19 is not so severe that i should get vaccinated	◯ Yes<br>◯ No
7. My immune system is so strong, it protects me aganist disease	◯ Yes<br>◯ No

8. Bad experience with similar vaccination	◯ Yes<br>◯ No

Convenience

1. Did not know where to get vaccination	◯ Yes<br>◯ No
2. Not possible to leave other work(home or office)	◯ Yes<br>◯ No
3. Long distance to the vaccination centre	◯ Yes<br>◯ No
4. Transport costs to the vaccination centre	◯ Yes<br>◯ No
5. Did not want to spend so much time at the vaccination centre	◯ Yes<br>◯ No

Calculation

1. Heard or read negative media	◯ Yes<br>◯ No
2. Did not know where to get good/reliable information	◯ Yes<br>◯ No
3. Distrust in government making decision in my best interest	◯ Yes<br>◯ No
4. Its important for me to fully understand COVID-19 vaccines before i get vaccinated	◯ Yes<br>◯ No
5. I closely consider whether COVID-19 vaccine is useful for me	◯ Yes<br>◯ No
6. I weigh the benefits and risks to make the best decision possible	◯ Yes<br>◯ No

Collective responsibility

1. When everyone is vaccinated, i don't have to get vaccinated too	◯ Yes<br>◯ No
2. When i am vaccinated, others are not protected	◯ Yes<br>◯ No
3. My friends/workmates didn't get vaccinated	◯ Yes<br>◯ No

Trust in source of information

What is your most trusted source of information about COVID-19 vaccines?	☐ Ministry of health<br>☐ Scientific journals<br>☐ Newspapers<br>☐ Online searching<br>☐ Social media<br>☐ Television<br>☐ Radios<br>☐ Others<br>(Tick all that apply)
What level of trust do you have for the above information about COVID-19 vaccines?	◯ Very trusted<br>◯ Moderately trusted<br>◯ Somewhat trusted<br>◯ Not trusted at all<br>(please select the most appropriate)
Would you recommend COVID-19 vaccines to others?	◯ Yes<br>◯ No
if no above, why	______________________________
